# Deconstruction of Vulnerability to Complex Diseases: Enhanced Effect Sizes and Power of Intermediate Phenotypes

**DOI:** 10.1100/tsw.2007.210

**Published:** 2007-11-02

**Authors:** David Goldman, Francesca Ducci

**Affiliations:** Laboratory of Neurogenetics, National Institute on Alcohol Abuse and Alcoholism, National Institutes of Health, Bethesda, MD, USA

**Keywords:** whole genome association, intermediate phenotype, endophenotype, alcoholism, effect size, complex diseases

## Abstract

The deconstruction of vulnerability to complex disease with the help of intermediate phenotypes, including the heritable and disease-associated endophenotypes, is a legacy of Henri Begleiter. Systematic searches for genes influencing complex disorders, including bipolar disorder, have recently been completed using whole genome association (WGA), identifying a series of validated loci. Using this information, it is possible to compare effect sizes of disease loci discovered in very large samples to the effect sizes of replicated functional loci determining intermediate phenotypes that are of essential interest in psychiatric disorders. It is shown that the genes influencing intermediate phenotypes tend to have a larger effect size. Furthermore, the WGA results reveal that the number of loci of large effect size for complex diseases is limited, and yet multiple functional loci have already been identified for intermediate phenotypes relevant to psychiatric diseases, and without the benefit of WGA.

Begleiter et al.[1] reported that amplitude of the P300 event-related potential was reduced in the alcohol-naïve, genetically at-risk, male offspring of alcoholics. This discovery indicated a disturbance of attentional processes in these boys and innate vulnerability due to impaired executive cognitive processes. Because alcoholism vulnerability is mediated by diverse mechanisms, identification of the P300 trait represented a key first step in a functionally based deconstruction of the illness of alcoholism and towards a more etiologically based redefinition of the disease. Subsequently, the low-amplitude P300 trait was also shown to be moderately heritable, thus establishing it as an endophenotype according to the definition of Gottesman and Gould[2].

Intermediate phenotypes can be defined as mechanism-related manifestations of complex phenotypes. Intermediate phenotypes that are heritable are called endophenotypes (see Fig. 1). Not all intermediate phenotypes useful in genetic studies are both heritable and disease associated, and some are neither[3]. For example, the intermediate phenotype of flushing is protective against alcoholism; rather than being more common in the disease, it is actually less common. Less obviously, but very frequently, intermediate phenotypes identify distinct subsets, sometimes very small, of the whole population of patients with a

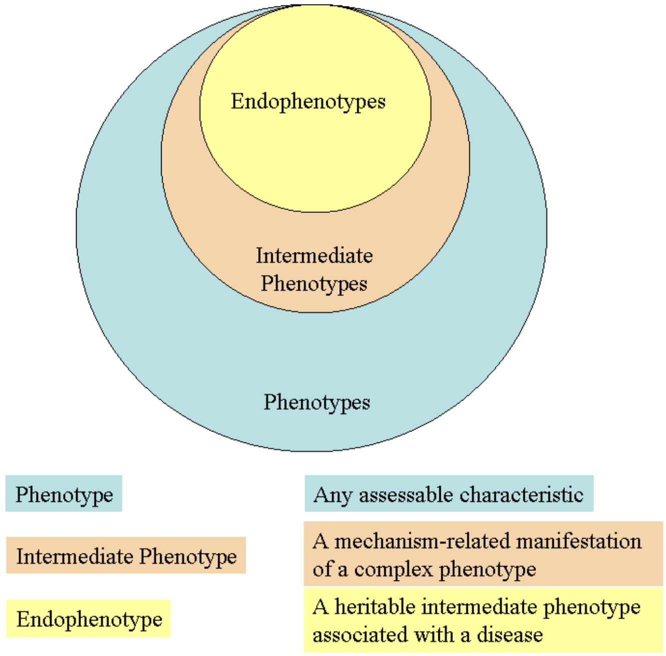

**FIGURE 1.** Definition of phenotype, intermediate phenotype, and endophenotype.

complex disease. In these instances, the intermediate phenotype is recognized to be disease associated only in retrospect, and after the intermediate phenotype has been used to redefine the disease. For example alcoholism, with a heritability of >0.50 and perhaps as high as 0.65[4], exhibits a higher heritability than P300 amplitude, which has a heritability of approximately 0.40[5]. However, the P300 trait identifies a more homogeneous population of alcoholics sharing a mechanism of vulnerability. Intermediate phenotypes also direct attention to particular genes and other etiologic factors, and enable directional predictions with functional alleles or these other etiologic factors. A noncomprehensive list of intermediate phenotypes for alcoholism is provided in Table 1. For several of these intermediate phenotypes, the involvement of particular genes was predicted on the basis of function and, in several instances, a functional locus has been replicably linked, as indicated.

The purpose of this paper is to compare the effect sizes of genes in complex diseases vs. those that have been discovered to influence intermediate phenotypes such as those listed. We would also like to begin to address the question of what fraction of the genes for complex diseases are discoverable using disease diagnosis alone. It is necessary to first observe that comprehensive lists of gene effects are available for neither. However, the recent completion of the Wellcome Trust WGA[6] study for seven complex diseases enables us to know with confidence all of the loci that are, on a genome-wide basis, capable of generating a significant signal in a comparison of 2,000 cases to 3,000 controls, and with a relatively dense (550,000 single nucleotide polymorphisms) genetic map. As shown in Table 2, the range of loci detected at the genome-wide confidence level ranged from a low of one for bipolar disorder to a high of seven for Crohn's disease. This study succeeded in detecting all previously replicated loci, so it is highly likely that few, if any, further loci of large effect will be discovered for any of them. The maximum genotype-specific effect sizes for these disease loci range from 1.5 to 18.5, with a median of 1.9. The fact that no locus with an effect size of less than 1.5 was detected even in this series of very large (2,000/3,000) case/control studies has very important implications, but here we are not concerned with the technical issue of power, but rather with the genetic architecture of the disease diagnosis, something that will not change even if sample size and marker density are increased. Even with these technical improvements, it is likely that additional loci detected will be of smaller effect, and extraordinarily large samples are required for loci of small effect.

**TABLE 1**
**Intermediate Phenotypes for Alcoholism**

| Domain | How Assessed | Heritability* | Locus |
|---|---|---|---|
| Diagnosis (e.g., dependence) | Diagnostic interview (e.g., SSAGA, SCID) | High | |
| Attention/dyscontrol | Low amplitude P300<br>Frontal cognitive tasks<br>Task-specific MRI<br>Questionnaires | Moderate | *COMT Val158Met (*rs4680)<br>*MAOA-LPR* |
| Pharmacokinetics | Flushing questionnaires<br>Metabolite levels | High | *ALDH2 Glu487Lys (*rs671)<br>*ADH1B His47Arg (*rs1229984) |
| Level of response | Alcohol challenge/clamp<br>Questionnaire (i.e., SRE) | High | |
| Treatment response | Response in clinical trials | ? | *OPRM1 Asn40Asp (*rs1799971) |
| Resting EEG | α, β resting EEG power | High | |
| Mesolimbic reward | Task-related MRI, PET | ? | |
| Stress/resiliency | Task-related MRI, PET<br>Endocrine responses<br>Questionnaires | ? | *HTTLPR (La, S, Lg)*<br>*COMT Val158Met (*rs4680)<br>*NPY* |
| Brain volume/structure | MRI structure | Region dependent | *BDNF Met66Val (*rs6265)<br>*MAOA-LPR*<br>*HTTLPR*<br>*COMT Val158Met (*rs4680) |

* High: >50%; Moderate: 30–40%.

The disease loci listed and detected by WGA represent important clues to etiology, but account for only a small portion of the total genetic variance in these diseases. For example, bipolar disorder has a heritability of 80–90% and a seven- to tenfold elevated recurrence risk in first-degree relatives of probands. The 16p12 locus listed is found in approximately one-half of bipolar cases and with an odds effect of approximately 2.1, accounts for perhaps 4% of the genetic risk for bipolar disorder. This leads to the conclusion that case/control samples for WGA need to be enlarged even further, but with rapidly diminishing returns, or that alternative strategies should be employed to better target genetic studies, as done before with candidate gene studies and studies on particular families, or to increase the effect size, as now will be discussed.

In contrast to the complex disease approach, the intermediate phenotype approach attempts to home in on mechanisms of vulnerability. For example, the activation of the brain stress axis constitutes a so-called “dark side” response to reinforcing drugs, leading to long-lasting and even lifelong vulnerability to stress-induced reinstatement of drug use, as well as cue-induced reinstatement. Functional loci at four genes, catechol-O-methyltransferase (COMT), the serotonin transporter (5-HTT), MAOA-LPR, and neuropeptide Y (NPY), have been linked to specific neural circuits associated with cognition and emotion. A functional missense variant in COMT, Val158Met (rs4680), partially accounts for variation in metabolic responses to working memory tasks in prefrontal cortex (PFC)[7], attentional control in cingulate cortex, and emotion in amygdala. The Met allele that leads to lower enzyme activity and predicts higher dopamine levels is associated with improved working memory and attentional control, but is also correlated with emotional response. Thus, the Met allele appears to be beneficial for cognitive function, at least under conditions of low or moderate stress, while the Val allele may benefit emotional resiliency. These results are consistent with higher pain/stress threshold and lower affective response to pain observed in Val carriers, and the greater ability of these individuals to activate brain opioid release after a pain/stress. In children, COMT genotypes strongly predict cognitive behavioral performance attributable to the prefrontal cortex, but not

**TABLE 2**
**Effect Sizes of Loci for Seven Diseases Analyzed by WGA in the Wellcome Trust Study**

| Phenotype | Locus | Heterozygote Odds Ratio | Homozygote Odds Ratio |
|---|---|---|---|
| Bipolar disease | 16p12 | 2.1 | 2.1 |
| Coronary artery disease | 9p21 | 1.5 | 1.9 |
| Crohn's disease | 1p31 | 1.4 | 1.9 |
| | 2q37 | 1.2 | 1.9 |
| | 3p21 | 1.1 | 1.8 |
| | 5p13 | 1.5 | 2.3 |
| | 5q33 | 1.5 | 1.9 |
| | 10q21 | 1.2 | 1.6 |
| | 10q24 | 1.2 | 1.6 |
| | 16q12 | 1.3 | 1.9 |
| | 18p11 | 1.3 | 2.0 |
| Rheumatoid arthritis (RA) | 1p13 | 2.0 | 3.3 |
| | 6 | 2.4 | 5.2 |
| Type 1 diabetes (T1D) | 1p13 | 1.8 | 5.2 |
| | 6 | 5.5 | 18.5 |
| | 12q13 | 1.3 | 1.8 |
| | 12q24 | 1.3 | 1.9 |
| | 16p13 | 1.2 | 1.6 |
| Type 2 diabetes | 6p22 | 1.2 | 2.2 |
| | 10q25 | 1.4 | 1.9 |
| | 16q12 | 1.3 | 1.6 |
| T1D, multilocus | 4q27 | 1.3 | 1.5 |
| | 12p13 | 1.6 | 1.5 |
| RA, sex differentiated | 7q32 | 1.4 | 1.6 |
| RA + T1D | 10p15 | 1.4 | 1.6 |

performance attributable to the dorsolateral prefrontal cortex. The functional polymorphism of the promoter region of the serotonin transporter gene (*HTTPLR*) has also been related to emotional processes. The short allele (S) that reduces the serotonin transporter gene *(SLC6A4)* transcription is associated with decreased amygdala activation by neutral stimuli, but enhanced activation by aversive or ambiguous cues[8] (Fig. 2). The S allele effect appears partly mediated by a decrease in connectivity to frontal and cingulate cortices, which modulate amygdala activity, and to structural changes, suggesting the possibility of effects on developing neurocircuitry in children.

The *HTTLPR* and *COMT* effects appear additive, presaging a future in which effects of various functional alleles are integrated with each other and with environmental predictors. A functional polymorphism (*MAOA-LPR*) located in the promoter region of monoamine oxidase A gene (*MAO*A) has been shown to moderate neuronal circuits associated with aggression and impulsivity. The low-activity variant at this locus predicted reduced limbic volume and hyper-reactivity of amygdala during emotional arousal, with diminished reactivity of regulatory prefrontal regions, compared with the high expression allele[9]. Consistently with these results, the low-activity variant has been reported to predict antisocial problems[10] and alcoholism[11].

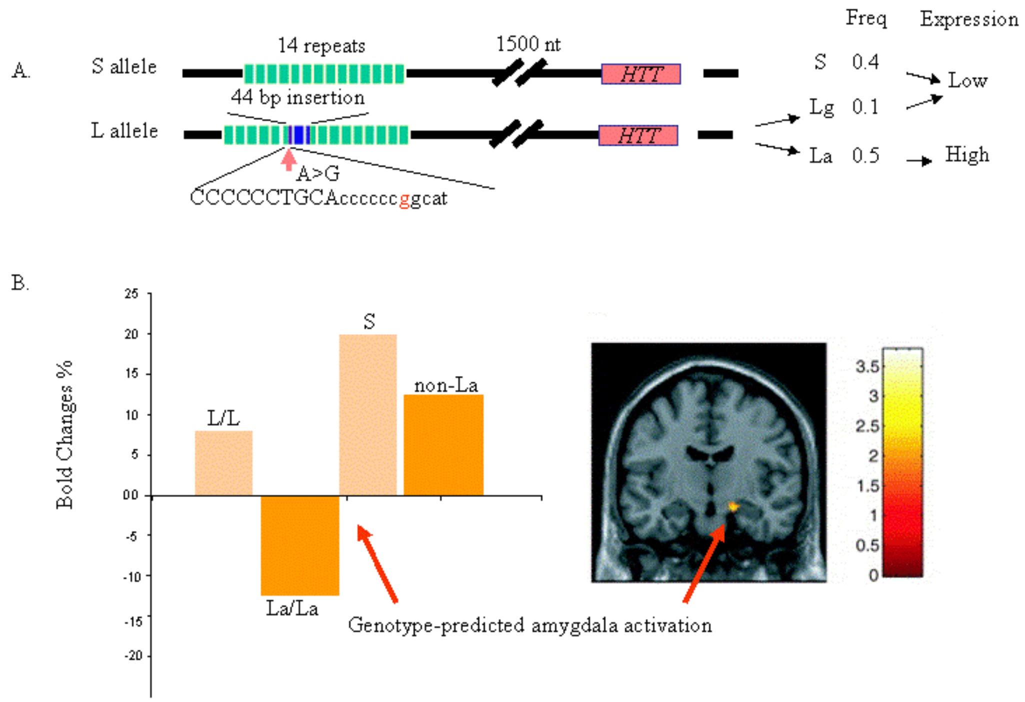

**FIGURE 2.** Serotonin transporter promoter polymorphism (*HTTLPR*) and relationship with amygdala activation during negative emotional stimuli. (A) A bi-allelic *HTTLPR* and a tri-allelic *HTTLPR*. Two forms of bi-allelic *HTTLPR*: long allele (L) and short allele (S). S allele decreases 5-HTT transcription and is associated with lower 5-HTT function. An additional A>G SNP is located on L allele of *HTTLPR*. $L_G$ decreases 5-HTT transcription similar to S allele. $L_A$ allele predicts greatest 5-HTT transcription and highest 5-HTT function. (B) Individuals with S allele increase amygdala activation, while individuals with $L_A$ allele have lowest activation in amygdala after negative emotional stimuli.

Effect sizes for functional loci in several intermediate phenotypes, including those just discussed, are listed in Table 3. It should be noted that a systematic scan for genes for any of these intermediate phenotypes has not been performed. Furthermore, the intermediate phenotype linkages detected and replicated thus far are necessarily of large effect size because they have been detected in relatively small datasets that are not powered to detect small effects. Nevertheless, and as shown in the table, this is a strategy that generates additional phenotypes on which functional alleles exert large effects, and yields immediate information on the mechanism of action of the functional allele. Genotype-specific effect sizes (Odds Ratio [OR]) reported in Table 3 have been computed for each intermediate phenotype, comparing the percentages of subjects within each genotype group who belong to the top third of the phenotype distribution vs. the percentage who belong to the bottom third of the trait distribution. This approach fails to capture all the information inherent in the quantitative trait, the adequacy of the approach depending on the varying precision with which each intermediate phenotype is estimated. Obviously genotype-specific ORs are much higher if more extreme tails of the quantitative distribution are compared, however in that case, sample sizes are reduced or some special phenotype- or genotype-based procedure would be required to enhance collection of individuals who are at the extremes of the distribution. Maximum genotype-dependent ORs calculated in this very conservative fashion range between 1.7 and 6.0. Of note, the lowest OR is for harm avoidance, a phenotype that can be considered a more complex phenotype than task-specific brain activation measured with functional MRI. Considering only fMRI studies, the median OR is 3.5, which is approximately twice the median locus-specific OR reported for the complex diseases in the Wellcome Trust WGA study.

**TABLE 3**
**Effect Sizes of Three Functional Loci on Different Intermediate Phenotypes for Psychiatric Diseases**

| Locus | Phenotypes | Subjects | N | Group Comparison | OR | Study |
|---|---|---|---|---|---|---|
| *COMTVal158Met* (rs4680 ) | WCST | Healthy controls | 55 | Val/Val vs. Met/Met | **6.0** | [7] |
| | | | | Val/Val vs. Val/Met | **1.7** | |
| | | Schizophrenics | 175 | Val/Val vs. Met/Met | **2.1** | |
| | | | | Val/Val vs. Val/Met | **1.6** | |
| *5-HTTLPR* | Amygdala activation | Healthy controls | 92 | ss vs. ll or ls | **3.9** | [8] |
| *5-HTTLPR* | Harm avoidance | Mixed | 5224 | ss vs. ll | **1.7** | [12] |
| | | | | ss vs. sl | **1.6** | |
| *MAOA-LPR* | Amygdala activation | Healthy controls | 77 | Low vs. High | **3.9** | [9] |
| | OCF activation | Healthy controls | 77 | Low vs. High | **3.1** | |
| | Hippocampus activation | Healthy controls | 44 | Low vs. High | **2.6** | |

## CONCLUSION

The small effect sizes of genes in complex diseases and limited number of loci detectable in large case/control datasets analyzed so far may presage an ultimate limit to the ability of this approach to access the genetic architecture of complex disease even with ever larger datasets, without preceding deconstruction of the complex phenotype with the help of intermediate phenotypes. Replicated linkages of functional loci to intermediate phenotypes in relatively small datasets indicate that WGA studies conducted on intermediate phenotypes, such as fMRI, are a powerful method to discover new genes that moderate brain activity. The large genotype-specific effect sizes detected by the functional locus/intermediate phenotype approach helps to explain the successes achieved with this method.

## References

1. Begleiter, H., Porjesz, B., Bihari, B., and Kissin, B. (1984) Event-related brain potentials in boys at risk for alcoholism. *Science* **225,** 1493–1496.
2. Gottesman, I.I. and Gould, T.D. (2003) The endophenotype concept in psychiatry: etymology and strategic intentions *Am. J. Psychiatry* **160,** 636–645.
3. Xu, K., Ernst, M., and Goldman, D. (2006) Imaging genomics applied to anxiety, stress response, and resiliency. *Neuroinformatics* **4,** 51–64.
4. Goldman, D., Oroszi, G., and Ducci, F. (2005) The genetics of addictions: uncovering the genes. *Nat. Rev. Genet*. **6,** 521–532.
5. Almasy, L., Porjesz, B., Blangero, J., Chorlian, D.B., O'Connor, S.J., Kuperman, S., Rohrbaugh, J., Bauer, L.O., Reich, T., Polich, J., and Begleiter, H. (1999) Heritability of event-related brain potentials in families with a history of alcoholism. *Am. J. Med. Genet*. **88,** 383–390.
6. Wellcome Trust Case Control Consortium (2007) Genome-wide association study of 14,000 cases of seven common diseases and 3,000 shared controls. *Nature* **447,** 661–678.
7. Egan, M.F., Goldberg, T.E., Kolachana, B.S., Callicott, J.H., Mazzanti, C.M., Straub, R.E., Goldman, D., and Weinberger, D.R. (2001) Effect of COMT Val108/158 Met genotype on frontal lobe function and risk for schizophrenia. *Proc. Natl. Acad. Sci. U. S. A.* **98,** 6917–6922.

8. Hariri, A.R., Drabant, E.M., Munoz, K.E., Kolachana, B.S., Mattay, V.S., Egan, M.F., and Weinberger, D.R. (2005) A susceptibility gene for affective disorders and the response of the human amygdala. *Arch. Gen. Psychiatry* **62,** 146–152.
9. Meyer-Lindenberg, A., Buckholtz, J.W., Kolachana, B., Hariri, A.R., Pezawas, L., Blasi, G., Wabnitz, A., Honea, R., Verchinski, B., Callicott, J.H., Egan, M., Mattay, V., and Weinberger, D.R. (2006) Neural mechanisms of genetic risk for impulsivity and violence in humans. *Proc. Natl. Acad. Sci. U. S. A.* **103,** 6269–6274.
10. Caspi, A., McClay, J., Moffitt, T.E., Mill, J., Martin, J., Craig, I.W., Taylor, A., and Poulton, R. (2002) Role of genotype in the cycle of violence in maltreated children. *Science* **297,** 851–854.
11. Ducci, F., Enoch, M.A., Hodgkinson, C., Xu, K., Catena, M., Robin, R.W., and Goldman, D. (2007) Interaction between a functional MAOA locus and childhood sexual abuse predicts alcoholism and antisocial personality disorder in adult women. *Mol. Psychiatry*. Epub ahead of print.
12. Sen, S., Burmeister, M., and Ghosh, D. (2004) Meta-analysis of the association between a serotonin transporter promoter polymorphism (5-HTTLPR) and anxiety-related personality traits. *Am. J. Med. Genet. B Neuropsychiatr. Genet*. **127,** 85–89.
